# Advances in Stabilization and Enrichment of Shallow Nitrogen-Vacancy Centers in Diamond for Biosensing and Spin-Polarization Transfer

**DOI:** 10.3390/bios13070691

**Published:** 2023-06-29

**Authors:** Federico Gorrini, Angelo Bifone

**Affiliations:** 1 Department of Molecular Biotechnology and Health Sciences, University of Torino, Via Nizza 52, 10126 Torino, TO, Italy; angelo.bifone@unito.it; 2 Center for Sustainable Future Technologies, Istituto Italiano di Tecnologia, Via Livorno 60, 10144 Torino, TO, Italy

**Keywords:** nanodiamonds, NV center, biosensing, charge stabilization

## Abstract

Negatively charged nitrogen-vacancy (NV^−^) centers in diamond have unique magneto-optical properties, such as high fluorescence, single-photon generation, millisecond-long coherence times, and the ability to initialize and read the spin state using purely optical means. This makes NV^−^ centers a powerful sensing tool for a range of applications, including magnetometry, electrometry, and thermometry. Biocompatible NV-rich nanodiamonds find application in cellular microscopy, nanoscopy, and in vivo imaging. NV^−^ centers can also detect electron spins, paramagnetic agents, and nuclear spins. Techniques have been developed to hyperpolarize ^14^N, ^15^N, and ^13^C nuclear spins, which could open up new perspectives in NMR and MRI. However, defects on the diamond surface, such as hydrogen, vacancies, and trapping states, can reduce the stability of NV^−^ in favor of the neutral form (NV^0^), which lacks the same properties. Laser irradiation can also lead to charge-state switching and a reduction in the number of NV^−^ centers. Efforts have been made to improve stability through diamond substrate doping, proper annealing and surface termination, laser irradiation, and electric or electrochemical tuning of the surface potential. This article discusses advances in the stabilization and enrichment of shallow NV^−^ ensembles, describing strategies for improving the quality of diamond devices for sensing and spin-polarization transfer applications. Selected applications in the field of biosensing are discussed in more depth.

## 1. Introduction

Negatively charged nitrogen-vacancy (NV^−^) centers are fluorescent defects in diamonds that have unique magneto-optical properties, such as single-photon generation [1,2,3,4], millisecond-long coherence times [5,6], and the possibility of initializing and reading the spin state via purely optical means. Owing to these features, NV^−^s have emerged in recent years as a powerful sensing tool for a number of applications, ranging from magnetometry [7,8] to electrometry [9,10] and thermometry [11,12]. Given their biocompatibility, which was demonstrated both in vitro and in vivo on several model organisms [13,14,15,16] NV-rich nanometric-sized diamonds (nanodiamonds (NDs)) find application in cellular microscopy, nanoscopy [17,18,19], and drug delivery [20,21], and as markers for in vivo imaging [22,23]. The detection of a few electron spins, as those of paramagnetic agents [24,25,26], and nuclear spins [27,28] was also demonstrated. Furthermore, techniques based on level anticrossing, cross-polarization, and the solid effect made it possible to hyperpolarize nitrogen defects and ^13^C nuclear spins in the diamond lattice up to a few percent [29,30], starting from the optically polarized electronic spins of NV^−^ centers. This might lead to the hyperpolarization of the nuclei outside the diamond lattice, opening new perspectives in NMR and MRI.

Most of these applications rely on the interactions between NV^−^ centers and the environment closest to the diamond surface. As a consequence, shallow NV^−^s have been engineered via low-energy nitrogen implantation (tens of kilo electron volts or less [31]) in macroscopic diamond crystals. Alternatively, nanodiamonds [32] and other nanostructures (e.g., nanopillars [33,34,35]) can be used to increase the surface area and expose shallow NVs.

Unfortunately, defects at the diamond surface such as hydrogen, vacancies, and trapping states [36,37,38] reduce the stability of the NV^−^ in favor of the neutral form of the center (NV^0^), which does not present the same optical and spin properties of NV^−^. Under certain power and wavelength conditions, laser irradiation can lead to charge state photoconversion and a reduction in the number of NV^−^ centers [39,40,41]. This often results in a degradation of the sensing properties of NV-based devices. For this reason, considerable effort has been devoted to understanding and improving the stability of NV^−^ centers either statically, via diamond substrate doping, annealing, and surface termination; or dynamically, via laser irradiation and electric or electrochemical tuning of the surface potential.

This article outlines the advances in the stabilization and enrichment of shallow NV^−^ ensembles using a variety of techniques and suggests strategies to improve the quality of the diamond devices for sensing and spin-polarization transfer applications. To the end, a few selected examples are discussed in more depth, with an emphasis on applications in biosensing.

## 2. Basic Physics of NV Centers

**Figure 1:**
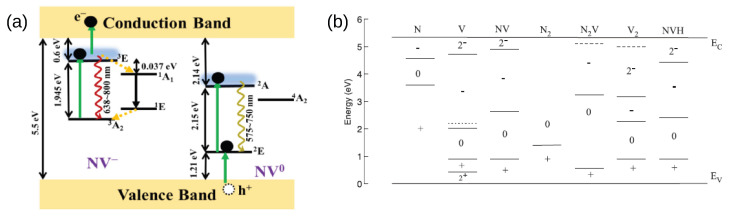
(**a**) The level scheme of NV${}^{-}$ and NV${}^{0}$ centers with the main transitions, including excitation, NV${}^{-}$ ionization via *e${}^{-}$* transition to the conduction band, and NV${}^{0}$ recharge via *h${}^{+}$* hole transfer to the valence band. Wavy lines represent radiative decay, and dashed lines represent the non-radiative decay involving singlet states. Reprinted with permission from Subedi et al. [59]. Copyright 2019 Optica Publishing Group. (**b**) The adiabatic charge transition levels for common defects in diamond. Substitutional nitrogen behaves as a donor, while vacancies are electron traps. Reprinted from Ref. [60], with permission.

The NV center is composed of a nitrogen atom coupled to a vacancy substituting two nearest-neighbor carbon atoms in the diamond lattice. It can be found in different charge states. The negative (NV^−^) and neutral (NV^0^) centers are the most abundant and studied, while the uncommon and optically inactive NV^+^ has only recently received some consideration [42,43,44]. Both NV^−^ and NV^0^ centers present energy levels within the wide diamond band gap of 5.5 eV (Figure 1a). The spin-1 NV^−^ center is characterized by a ground state (a spin triplet ^3^A_2_), an excited state (^3^E), and additional intermediate singlet states (^1^A_1_, ^1^E). It displays fluorescence, with an absorption band in the visible range [45] and an emission spectrum composed of a zero-phonon line (ZPL) at 637 nm (equivalently, 1.945 eV) and an associated phonon sideband extending to the near-infrared range [46]. The peculiarity of the NV^−^ center lies in its laser-induced optical spin-polarization and readout of the ground state. This depends on the existence of a non-radiative transition from the excited to the ground state through singlet states. Because of this mechanism, a spin polarization exceeding 80% can be obtained via pure optical irradiation [47]. In the ground-state manifold, the s = ±1 levels are raised by 2.87 GHz relative to the s = 0 level due to the zero-field splitting [48]. Additional couplings to diamond strain fields, external electric and magnetic fields, and electronic or nuclear spins (mostly nitrogen and ^13^C) affect the energy levels of the NV^−^ center [49]. It was shown that these differences can be evaluated using either continuous or pulsed techniques [50,51,52,53], such as optically detected magnetic resonance (ODMR), free induction decay, and spin echo, leading to the quantitative measurement of these interactions.

Similarly, the neutral NV^0^ center has an excited state (^2^A) separated from the ground state (^2^E) by 2.156 eV [54] and can be excited with visible light from blue to yellow. The defect, which is also active in cathodoluminescence, has a ZPL at 575 nm and an associated vibronic sideband up to 750 nm [55]. Despite its similarity with NV^−^ in terms of energy of excitation and fluorescence, the NV^0^ has no interesting magneto-optic properties: the absence of a laser-induced spin polarization limits its utility for sensing. An exception is provided by the measurement of pressure, strain fields, and temperature through ZPL analyses [56,57,58].

## 3. Charge Stabilization by Doping and Surface Termination

The two centers were first identified by their spectral properties, absorption, and fluorescence (FL), but it was only with the work of Mita [55] and Iakoubovskii [61] that they were recognized as two charge states of the same structural defect. As NV^0^ and NV^−^ contain five and six electrons, respectively, one could expect some charge-state transition in the form of a single electron capture or release. Indeed, these phenomena were observed to be either spontaneous or induced, temporary or permanent, depending on several parameters, which include the processing of diamond (synthesis, annealing, and irradiation with electrons or ions); surface passivation and termination; depth of the NV center; and, finally, the wavelength, duration, and power of laser irradiation. Additionally, the presence of other defects in the diamond (Figure 1b) modifies the stability of NV center states. The following discussion gives an overview of these processes and mostly focuses on shallow NV centers, those typically employed for sensing and NMR-based schemes.

**Figure 2:**
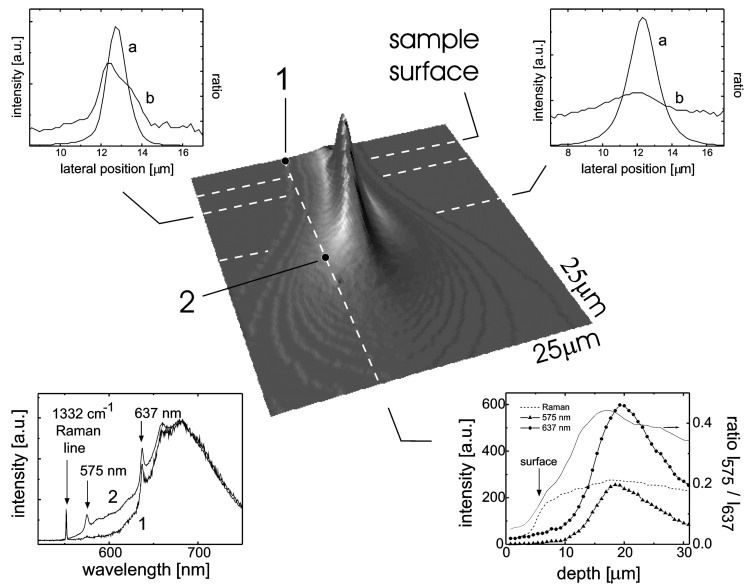
Three-dimensional fluorescence imaging of NV centers (at the center). The two top panels plot the fluorescence intensity (line a) and the relative amount of NV${}^{0}$ centers (line b) along the dotted lines in the central figure. The fraction of NV${}^{0}$ increases with irradiation dose and content of NV centers. The right bottom panel shows a vertical scan of NV${}^{-}$ FL, NV${}^{0}$ FL, their ratio, and the diamond Raman line. Fluorescence taken at points 1 and 2 is shown in the bottom left panel, indicating again a higher fraction of NV${}^{0}$ in the region of more severe irradiation damage. Reprinted from Ref. [71], with permission.

Two techniques are widely used to create NV centers. The first is irradiation with electrons [62] or heavier ions [63,64] of a nitrogen-rich diamond, followed by annealing at a temperature in excess of 600 °C [65]. Irradiation produces vacancies that become mobile during annealing and recombine with native substitutional nitrogen atoms to produce NV centers. This is often an efficient strategy for the production of high-density NV centers [62]. The second technique relies on the direct implantation of nitrogen (single ions or ionized molecules) on a pure, type IIa diamond crystal, followed by high-temperature annealing [66]. By regulating the fluence of nitrogen ions [67], very dilute ensembles of NV centers can be obtained, enabling single NV manipulation. Moreover, the depth of implanted nitrogen and, in turn, of NV centers is determined by the energy of the implanted nitrogen ions. This enables the synthesis of ultra-shallow, NV centers that are single-digit nanometer deep [31,68,69]. While these implantation techniques have been routinely applied, they also have downsides. Waldermann [70] used He^+^ ions to create vacancies in type Ib diamond for micrometer-deep NV centers. Evaluating the fluorescence emission, they observed increasing NV^−^ and NV^0^ formation with He fluence up to 10^15^ ions/cm^2^, after which both charge states signals dropped steadily. Additionally, approaching 10^17^ ions /cm^2^, the majority of NV centers are in the neutral form. A similar effect was reported by Martin [71] with electron irradiation of type Ib diamond (Figure 2). This promoted the creation of NV centers in the region of more severe radiation damage because of the availability of vacancies during the annealing phase. Unfortunately, this region also contains the largest fraction of NV^0^ over the total number of NVs. Therefore, a high radiation dose promotes the formation of NV centers, but can compromise the stability of the NV^−^ by favoring the neutral state.

**Figure 3:**
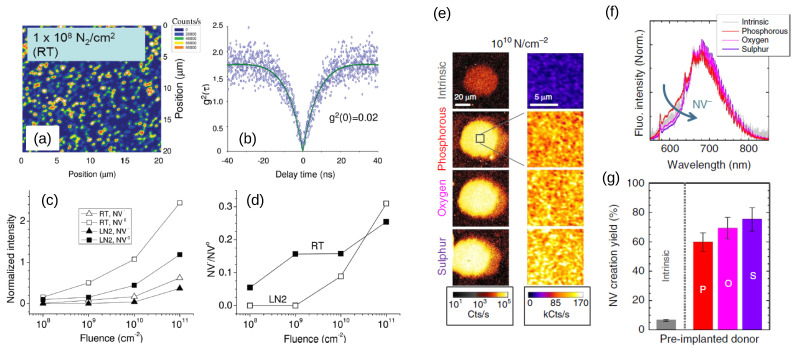
Confocal spectrum of NV center implanted in IIa diamond to a fluence of 10${}^{8}$ ${}^{15}$N${}_{2}$ cm${}^{-2}$ at RT (**a**) and second correlation function showing single-center emission (**b**). The fraction of NV${}^{0}$ compared with NV${}^{-}$ is large (**c**,**d**) for implantation at both room temperature (RT) and liquid nitrogen (LN2) conditions, at all the inspected fluences of implanted nitrogen and even after annealing for 1 h at 800 °C. Reprinted from Ref. [67], with permission. Nevertheless, the stability of NV${}^{-}$ can be augmented via donor implantation. Nitrogen doping and annealing in preimplanted diamond with phosphorous, oxygen, and sulfur (following annealing at 1200 °C) results in intense confocal-detected fluorescence (**e**); a higher fraction of NV${}^{-}$, as shown by normalized FL spectra (**f**); and an overall increase in NV creation yield by an order of magnitude (**g**) compared with the intrinsic, undoped diamond. Readapted with permission from Lühmann et al. [80]. Copyright 2019 Springer Nature.

The direct implantation of nitrogen followed by annealing also results in a large, often predominant fraction of NV^0^ [66,67,72] (Figure 3a–d). Additionally, the NV^−^ centers obtained via this method are characterized by charge conversion to NV^0^ and reduced photostability [73,74]. High-temperature annealing (above 700 °C) shows limited effects on the stabilization of NV^−^ compared with the postirradiation conditions [62,67,75]. The reduced NV^−^ stability after ion/electron implantation can be readily explained: a negative NV center requires an additional electron (a total of six), which is usually provided by substitutional nitrogen. However, implantation creates a large fraction of vacancies and trapping states [31], often outnumbering the available nitrogen ions. Single vacancies present a negative state that is lower in energy compared with NV^−^ [60]. If these trap states do no annihilate or recombine during annealing, they can deplete the nitrogen donors of electrons and favor NV^0^ stability. Increasing the dose of implanted nitrogen is not always viable, also considering that NV^−^ coherence times decrease with nitrogen concentration [76]. Nevertheless, the adverse effect of vacancies can be contrasted by doping diamonds with electron donors, which shift the Fermi level upward with respect to the valence band maximum, an effect that can be observed with ultraviolet photoelectron spectroscopy (UPS) [77]. Indeed, in the case of low-density nitrogen implant samples, phosphorous [78,79], as well as oxygen and sulfur [80], can be efficiently incorporated into the diamond, resulting in higher NV^−^ quality (Figure 3e–g). Despite recent progress, these results are not readily reproduced in high-N implant densities, and more research is required to stabilize dense NV ensembles through substrate doping.

**Figure 4:**
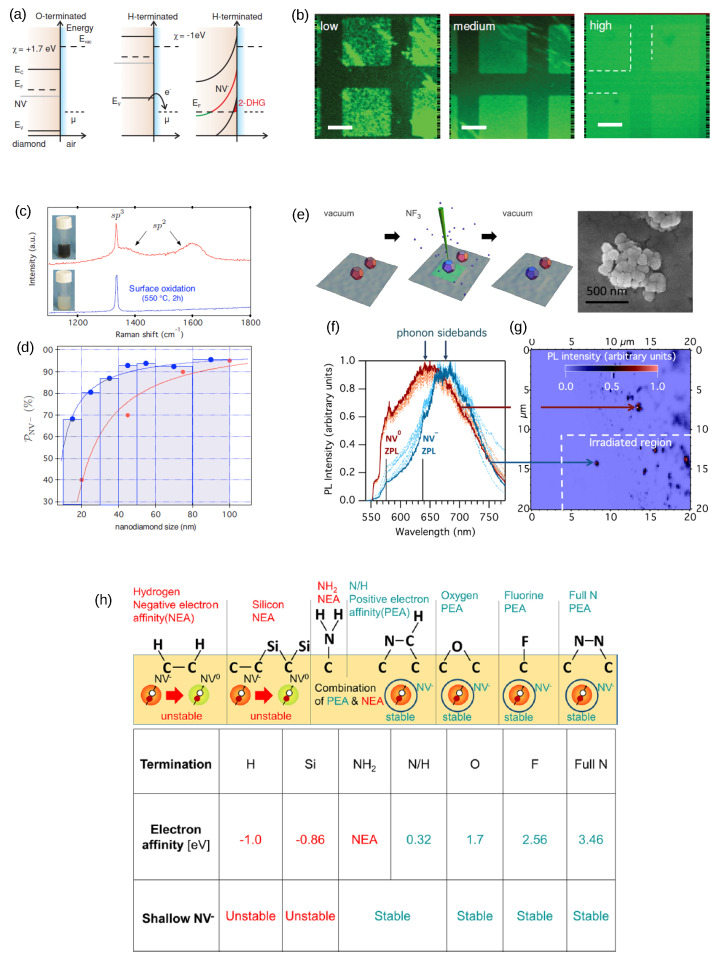
Energy band schematic of diamond (**a**). The 1.7 eV positive electron affinity with oxygen termination stabilizes the NV${}^{-}$ centers. On the contrary, hydrogen results in −1.0 eV electron affinity, transfer of electrons outside the surface, and the creation of a two-dimensional hole gas (2-DHG). The red line represents the NV${}^{-}$ instability while crossing the Fermi level close to the surface. (**b**) The FL contrast between hydrogen- and oxygen-terminated diamond (dark regions and bright squares, respectively) follows the concentration of NV${}^{-}$ centers. The contrast weakens with increasing nitrogen implantation dose, following a higher amount of donor impurities that reduce the negative impact of hydrogen at the surface. From Ref. [36], reprinted with permission. Surface oxidation (**c**) of nanodiamonds favors stabilization of NV${}^{-}$, as evidenced by the reduction in the nondiamond sp${}^{2}$ signal and the lightening of the color of the solution. After this treatment, a NV${}^{-}$ fraction of up to 70% of the total can be obtained, even for small 10 nm particles (**d**). The red and blue colors correspond to the nonoxidized and oxidized NDs, respectively. Reprinted from ref. [93], with permission. A similar result was obtained via the selective fluorination of clustered NDs using electron irradiation in NF${}_{3}$ gas (**e**). Electron irradiation results in clusters of surface-fluorinated nanodiamonds. The PL of virgin, H-terminated (red curves), and fluorine-treated NDs (blue curves) is plotted in (**f**) together with the FL map separating the irradiated and the unirradiated regions (**g**). Reprinted from ref. [94], with permission. Types of terminations with surface electron affinity and stability of shallow NV${}^{-}$ are summarized in (**h**). Reprinted with permission from Kawai et al. [90]. Copyright 2019 ACS American Chemical Society.

The picture is more complicated for ultra-shallow NV centers (<20 nm), where radiation damage [64] and surface termination have a direct effect on NV charge state stability. In fact, it was demonstrated that the positive or negative electron affinity of the surface determines the dominant charge state of NVs. Hydrogenation causes a negative electron affinity of −1.0 eV [81,82,83], an upward band bending, and the creation of a 2D hole conductive layer induced by atmospheric adsorbates behaving as acceptors [84]. Although dependent on the surface ion concentration [85], upward band bending in general favors NV^0^ centers (Figure 4a,b). NV^−^ is unstable with silicon termination as well. In addition to band bending, surface termination may create surface acceptor states (gap states), which represent an important concern for NV^−^ stability. Using UPS and X-ray absorption spectroscopy, two gap states have been identified and experimentally observed: the primal sp^2^ defect [86] and one originating from a highly disordered mixture of ether, alcohol, and carbonyl groups [87]. Unfortunately, electronic-trapping gap states might also be deleterious for spin coherence, providing a source of magnetic and electric noise. Despite the need for further research, there is evidence that oxygenation [36,88], fluorination [69,89], or nitrogenation [90] of the surface increases both the total yield of NV and the abundance of the negative states (Figure 4). Moreover, the successful stabilization of NV^−^ with a glycerol coating [91] and graphene junctions [92] has been recently reported.

Turning to nanodiamonds [95,96,97], all previous considerations hold, but one also should account for a large heterogeneity in shape (quantified by the aspect ratio) and size (resulting in different surface-to-volume ratios). Several techniques for the production of nanodiamonds have been developed [98], including detonation [99], chemical vapor deposition [100], diamond-anvils-cell synthesis [101], high-power pulsed-laser carbon melting [102,103], and top-down processes such as ball-milling of microdiamonds [104]. Depending on the application, a specific synthesis technique must be chosen as it determines the size, degree of crystallinity, concentration of dopants, and surface purity of the NDs. Surface noise is another factor that has a direct impact on NV^−^ stability. In fact, as highlighted by Rondin [93], the unfavorable effect of surface noise on NV^−^ stabilization is stronger for smaller NDs. This heterogeneity does not exclusively affect the charge state ratio but also results in a larger variability in the fluorescence intensity and the excited state lifetime of both NV^−^ and NV^0^ [105,106]. Again, some strategies can help improve NV^−^ stability (Figure 4c–h), such as a high initial nitrogen content (in the order of hundreds of parts per million) [104], optimized ion implantation [106], optimized annealing [107], and functionalization of the surface with oxygen and fluorine [94,108,109]. Importantly, tailoring the surface chemistry of NDs [95,105,110] extends the possible applications with respect to macroscopic diamond crystals. For instance, functionalization of the surface can favor the endocytosis of NDs, which is a less invasive technique than electroporation for cellular permeabilization or the use of microneedles [16]. Despite their increasing success as biosensors (as discussed in the Application Section), there are still several issues that need to be addressed for the widespread use of NDs, especially those smaller than 10 nm. Firstly, the cytotoxicity associated with nondiamond surface carbon needs to be taken into account, and techniques for surface cleaning should be further refined [111]. Secondly, measures must be taken to prevent agglomeration and surface-induced charge instability, particularly when it comes to single-molecule labeling or imaging of the smallest cellular compartments [99]. Thirdly, because the number of NV^−^ decreases as the size of NDs decreases, a significant portion of the smallest NDs (<10 nm) does not exhibit fluorescence from NV centers [95]. Hence, improving synthesis, implantation, and annealing processes is crucial to obtain reliable bioprobes.

## 4. Laser-Induced Charge Switching

**Figure 5:**
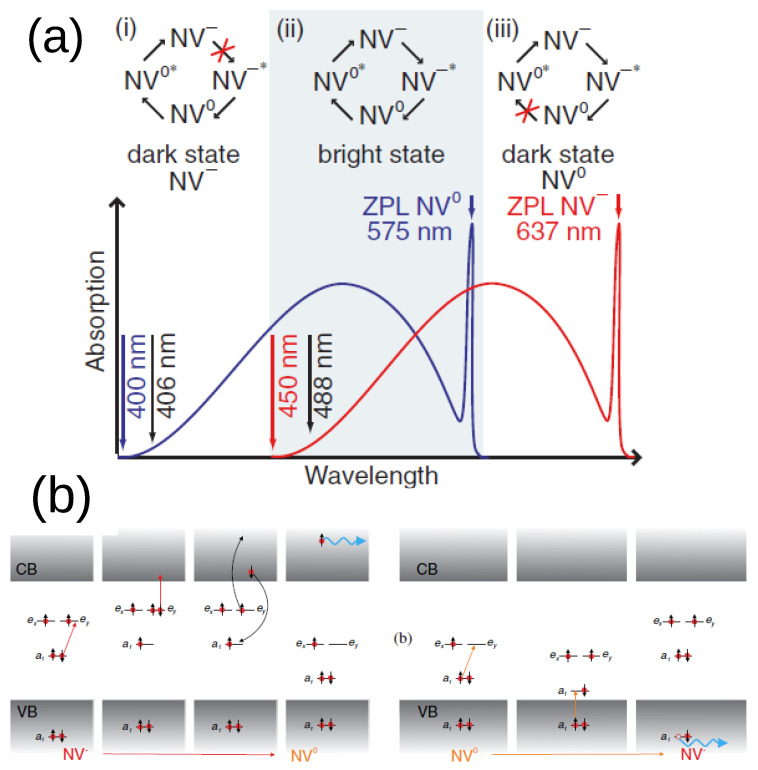
(**a**) Pictorial representation of the low-temperature absorption spectra of NV${}^{-}$ (red) and NV${}^{0}$ (blue). At the top, the relevant switching cycles are outlined. Arrows indicate laser-induced transition toward the excited states (denoted by the asterisk) or to a different charge state. A wavelength included in the gray-shaded region is able to excite and photoconvert both charge states; continuous charge switching is then ensured. In the white regions, the wavelength is not exciting either the NV${}^{-}$ (for λ < 450 nm) or the NV${}^{0}$ (for λ > 575 nm). In these cases, the photoconversion loop is broken (red crosses), and a preferential population of a particular charge state occurs. Reprinted from Ref. [40], with permission. (**b**) Schematic picture of charge conversion among neutral and negative NVs. NV${}^{-}$ to NV${}^{0}$ conversion involves two photons to detach an electron from the defect. The inverse process, NV${}^{0}$ to NV${}^{-}$ conversion, also occurs in two steps: an electron is excited to the *e* orbital from the *a${}_{1}$* orbital in the band gap. The vacant place is then occupied by a second electron transferred from the deep-lying *a${}_{1}$* orbital. The hole migrates away from the newly formed NV${}^{-}$ center. From Ref. [115], reprinted with permission.

A different strategy to modulate the charge state of NVs is through optical irradiation. The photochromism of NV centers has been demonstrated in a number of papers. Both conversions from NV^−^ to NV^0^ (ionization) and from NV^0^ to NV^−^ (recharge) have been shown to occur under laser irradiation and in the dark [37,39,74,112]. This apparent complexity is due to several factors [40,41,113]: first, the type of dominating defects, such as interstitial nitrogen for type Ib diamond or impurities at the surface; second, the wavelength of the laser, which can excite either one or both of the charge states according to their absorption spectra (Figure 5a); third, the intensity of laser irradiation. Thus, starting from an initial balance between NV${}^{0/-}$ centers, continuous laser irradiation creates a new steady-state equilibrium, corresponding to a different ratio of the NV${}^{0/-}$ centers. Typically, after switching the laser off, the system relaxes toward a third condition of equilibrium, again influenced by the type of defects in the lattice. Because laser irradiation can also modify the spin states of the NV^−^, the charge and spin states are, in general, simultaneously initialized and read out, unless decoupling techniques are conceived [114]. In the following section, these processes are described in more detail.

A sufficiently intense laser with a wavelength above 575 nm selectively depopulates the NV^−^ centers, since it has enough energy to first excite and then ionize NV^−^, without being absorbed by the NV^0^ centers (Figure 5a). Conversely, a blue laser (<450 nm approximately) depopulates the NV^0^, as the neutral charge state has an absorption line in that range, unlike the negative state. A well-accepted description of the recharge mechanism is via NV^0^ excitation followed by hole capture by the valence band (Figure 5b). Thus, both photoconversion mechanisms are two-step processes [40,115]. In the x 450–575 nm exclusively, a laser is able to excite and ionize both centers, and a steady-state equilibrium is set between the two charge states depending on the radiation intensity [113]. This equilibrium is summarized by the NV populations [41]:

(1) $$\begin{array}{c} P_{NV^{-}}=\frac{k_{r}}{k_{r}+k_{i}}\\ P_{NV^{0}}=\frac{k_{i}}{k_{r}+k_{i}} \end{array}$$

where $k_{i}$ and $k_{r}$ are the ionization and recharge rates, respectively, and depend on laser wavelength and intensity. In the case of a single NV center, under 637 nm red laser irradiation (centered at the NV^−^ ZPL), $k_{r}$ is almost zero, and a high NV^0^ state fidelity of 95% can be reached [116]. On the contrary, the commonly employed 532 nm green laser cannot initialize the NV^−^ population beyond 80% [41,116]. Nevertheless, we note that charge-state conversion can also be induced by simultaneous, two-laser irradiation, typically a 532 nm green and a 1064 nm IR laser [117,118,119] or in the form of pure-IR multiphoton absorption (with two or three photons involved in the process) [120,121]. This multicolor modulation produces a pronounced improvement (above 90%) in the NV^−^ initialization fidelity, as recently reported [122].

**Figure 6:**
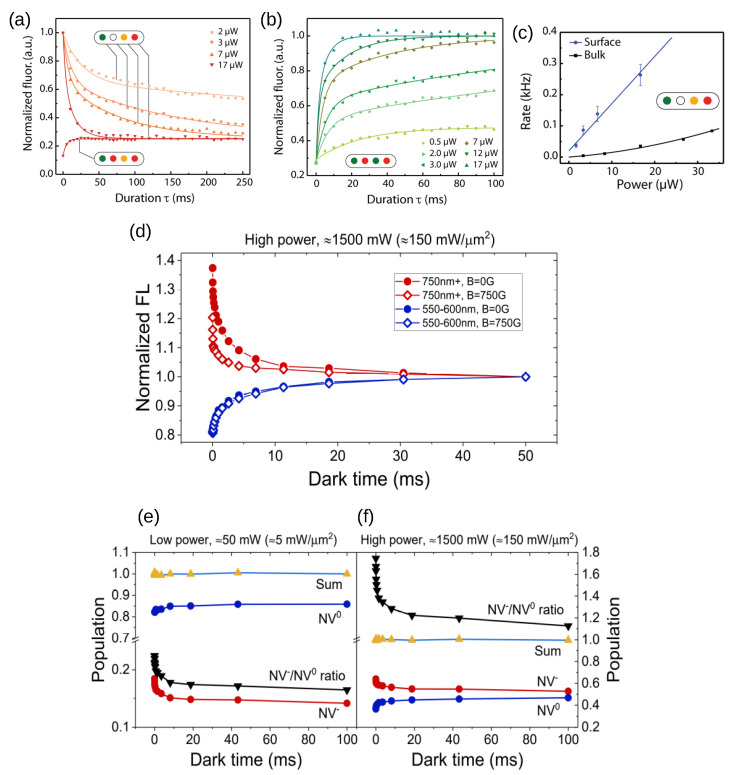
Several pulse sequences are employed to measure photoconversion. In (**a**), the ionization rate of shallow (<10 nm) NV${}^{-}$ is measured as a function of orange 594 nm laser power and duration (in ms). The colored dots denote the sequence of wavelengths of the pulsed lasers: a green laser initializes the NVs into the negative state, while the orange laser (of variable duration and power) reduces the NV fluorescence via photoionization to the neutral state. The red laser is used for read out. In (**b**), the recharge rate of NV${}^{-}$ is measured as a function of green 532 nm laser power and duration. The first two lasers (green and red) prepare the NV charge into the neutral state; the third laser (532 nm), of variable power and duration, restores the NV${}^{-}$ charge state, accompanying an increase in FL. The last pulse with a red laser is used to read out the charge state. Interestingly, ionization and recharge rates are quadratic with laser power for bulk NV but linear for shallow NV centers, suggesting a single-photon-mediated photoconversion mechanism (**c**). Adapted with permission from Dhomkar et al. [112]. Copyright 2018 American Chemical Society. The spin and charge dynamics can be decoupled using a magnetic field that mixes the spin statistics and quenches the FL (**d**). The signal arising from NV${}^{0}$ (blue lines) does not include any spin-related effect and is unaltered by the magnetic field. On the contrary, the quenching of FL is evident for the NV${}^{-}$ centers (red curves), suppressing the spin dynamics and revealing the charge dynamics. The NV${}^{0}$ and NV${}^{-}$ charge dynamics are complementary as they proceed in the opposite direction and can be isolated with a proper set of filters. The populations of NV${}^{-}$ and NV${}^{0}$ can be normalized and plotted together with their sum and ratio (**e**,**f**): it can be seen that after high-power laser irradiation, the NV${}^{-}$/NV${}^{0}$ ratio remains large even after 100 ms. A possible explanation involves depleting the acceptor states by filling them with electrons released by substitutional nitrogen atoms, therefore stabilizing the negative NVs. Reprinted with permission from Gorrini et al. [35]. Copyright 2021 American Chemical Society.

Finally, we see that the stability of NV${}^{0/-}$ centers depends on the availability of electron donors and traps in the surroundings, which might be different before and after laser irradiation. Thus, in the bulk, most of the NV centers are initially negatively charged, and laser irradiation, through photoionization, can shift the equilibrium toward NV^0^ centers. After switching the laser off, the equilibrium is restored via a back conversion of NV^0^ to NV^−^, supposedly via electron tunneling [123]. Completely different is the case of NVs close to the surface, where the presence of defects, such as vacancies and surface acceptors, often makes NV^0^ the preferential state. Here, a laser can recharge NV^0^ and increase the fraction of NV^−^ [35,37,38,74,112,113,118] (Figure 6a,b). Depending on the number of defects, the ionization/recharge rates can turn linear with laser power (Figure 6c) [35,112,114], denoting a single-photon process underlying the photoconversion mechanism. A common trait of shallow NV^−^ centers is the tendency to spontaneously convert back to NV^0^s in the dark (Figure 6d–f). For NV^−^ ensembles, the FL intensity follows a stretched exponential law:

(2) $$I\left(t\right)=I_{eq}\left[1+A\exp{\left(-\frac{t}{T_{i}}\right)}^{n}\right]$$

where *I*${}_{eq}$ is the equilibrium FL, *A* is an amplitude coefficient, *T*${}_{i}$ is the characteristic time of ionization, and *n* is a stretching coefficient, reflecting the collective emission from an ensemble of NVs. For single-NV centers, double-exponential and even single-exponential laws have been reported, suggesting NV^−^ electron transfer toward a single preferential defect [37,112,124]. Importantly, ionization in the dark depends on the illumination history, and long-lived NV^−^ states can be created [35,37,112] by properly tuning the laser parameters. It was theoretically proposed [119] and then conclusively demonstrated [35] that a high-power green laser can increase the fraction of the negative state compared with the neutral one in the case of NV ensembles. Quantification of the photoconversion process was achieved by quenching spin dynamics with a magnetic field [35,114] (Figure 6d–f). These long-lived charged states could be manipulated on time scales exceeding the duration of the protocols employed in magnetometry, in vivo fluorescence detection, and spin polarization transfer. Interestingly, the origin of NV^−^ stabilization was attributed to laser-induced filling of acceptor states (traps), following nitrogen ionization and an excess of electrons populating the conduction band. Thus, laser initialization could be a complementary technique to surface termination as a method to produce ensembles of shallow NV^−^ with sufficiently long lifetimes for sensing and polarization transfer applications.

## 5. Applications

In this section, we illustrate the effects of charge dynamics in some experimental contexts. While charge-state conversion opens new possibilities in biosensing and microscopy, in other cases, it is detrimental and must be suppressed to the highest possible degree.

### 5.1. Charge-Conversion-Based Applications

**Figure 7:**
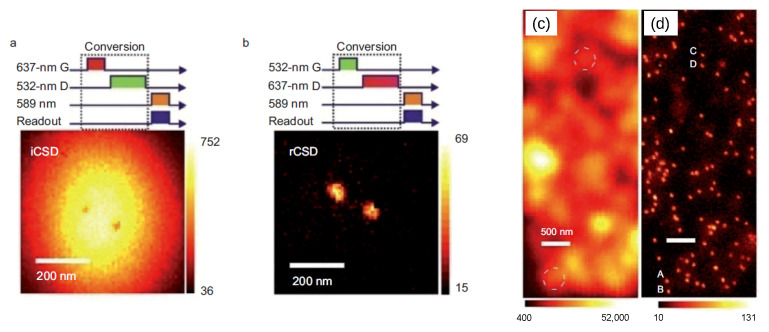
Super-resolution imaging lead by charge photoconversion. In (**a**), the irradiation with red Gaussian-shaped (G) and green doughnut-shaped (D) laser beams emphasizes the NV${}^{-}$ emission, except in a small central region, where the NV centers are in the dark neutral state (the two photobleached spots at the center). In (**b**), a similar sequence with inverted laser wavelengths suppresses the FL everywhere (all the NV are neutral), except at the center (the two bright spots). By tuning the intensity and duration of the doughnut pulse, it is possible to improve the spatial resolution: this is evident when comparing traditional confocal imaging (**c**) with the present technique (**d**). For instance, points A and B, or C and D cannot be resolved individually with a confocal microscope, while super-resolution imaging allows extraction of fluorescence and ODMR signals. Readapted with permission from Chen et al. [18]. Copyright 2015 Springer Nature.

Charge photoconversion is pivotal for diffraction-unlimited nanoscopy [17,18,19]. This method uses two different lasers (typically green and red) to selectively initialize either charge state with high fidelity (Figure 7a,b). The first laser has a Gaussian profile and prepares NV centers in a particular state (for instance, the negative one, which is brighter, as shown in Figure 7b). The other laser beam is doughnut-shaped, with an intensity profile approaching zero at the center. Using this doughnut beam, NVs can be photoconverted into a different charge state [17] (in this example, the neutral darker state) everywhere except in a small region at the center. Theoretically, by tailoring laser power and duration, this central region can be indefinitely shrunk. In this way, an optical resolution of 4.1 nm was demonstrated [18], well below the diffraction limit (Figure 7c,d). A similar technique, named stimulated emission depletion (STED) nanoscopy [125], has been tested with NDs internalized in cells, with a similar diffraction-unlimited resolution [126,127]. However, we suggest that charge-conversion-based super-resolution imaging might be more suitable for biological materials since it requires a lower laser power than STED. Additionally, a combination of diffraction unlimited nanoscopy with SEM/TEM microscopy (named correlative light electron microscopy (CLEM)) [128,129] or with α-particles iono-nanoscopy [130] has also been applied to single cells, with promising results. All these techniques could enable the diamond-assisted super-resolution imaging of bioassays and nanophotonic investigations.

**Figure 8:**
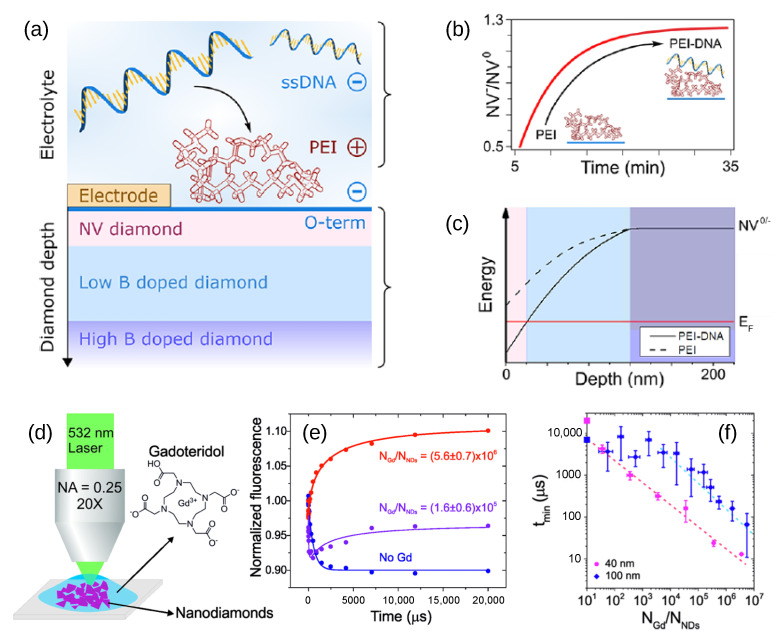
NV centers can serve as nanoprobes for the detection of magnetic and electric fields. A sophisticated setup constituted by an oxygen-terminated boron-doped single-crystal diamond (**a**) can initialize the NV center into the neutral state due to a combination of polyethylenimine (PEI) molecules electrostatically attached to the surface and an electrode to tune the electrostatic potential. When single-stranded DNA molecules adhere to the PEI layer, the surface electrostatic potential changes, and the NV centers turn to negative: it is possible to track this change in real time (**b**). In (**c**), the energy of the NV defects compared with Fermi energy (red line) is displayed for the two cases of PEI and PEI-DNA surface functionalization. In the pink-colored region, close to the oxygenated surface, the charge state of the NV centers depends on the presence of PEI or PEI-DNA molecules attached to the surface (dashed and solid black lines, respectively). Reprinted from Krečmarová et al. with permission [131]. Copyright 2021 American Chemical Society. When NDs are suspended with paramagnetic molecules (gadoteridol, in the figure), (**d**) the NV${}^{-}$ spin relaxes quicker and uncovers the signal due to charge dynamics, which proceeds in the opposite direction (**e**). The composition of spin relaxation and charge recovery produces a nonmonotonic trend that depends on the concentration of paramagnetic molecules per diamond nanoparticle. The net effect is a shift of the FL minimum, located at t${}_{min}$, at shorter times (**f**). Readapted with permission from Gorrini et al. [26]. Copyright 2019 American Chemical Society.

NV charge-state conversion induced by external agents may be exploited for sensing electrically charged molecules adsorbed at the diamond surface [131] or, for NDs, the pH and temperature variations in the host solution [132,133]. A simpler framework only considers the NV charge-state modification through the shift in the fluorescence spectrum, without accounting for the NV^−^ spin dynamics. In fact, it has been demonstrated that a polymer shell coating the nanodiamonds can swell or collapse depending on temperature variations or because of deprotonation of the carboxylate groups following a change in pH [132,133]. This determines a reversible change in the electric potential close to the ND surface, resulting in an NV charge-state conversion, detectable via optical means. The same concept was applied to a highly engineered single-crystal diamond equipped with a microfluidic cell and surface electrodes (Figure 8a–c): here, the surface potential was modified via the absorption of negative DNA molecules, capable of inducing a measurable charge switching of shallow NV centers [131].

The intrinsic sensitivity of NV charge dynamics to electric fields, either produced by external charges or by electronic nanodevices, might also enable NV-based scanning electrometry [134]. In fact, the traditional ODMR schemes often fail to measure electric fields via the Stark effect because of the poor coupling with NV spins [135], which results in a hardly detectable shift in their electronic levels (often <0.1 MHz). Technologies based on charge switching instead of spin dynamics might dramatically increase sensitivity.

Recently, a framework to simultaneously describe charge and spin dynamics has been proposed [26,124,136]. It was shown that the rates relevant for spins (rates of spin polarization and relaxation) and charges (rates of NV^−^ ionization and recombination in the dark) are interdependent, such that spin and charge dynamics are coupled mechanisms. The presence of fluctuating magnetic fields, such as those originating from paramagnetic molecules or superparamagnetic nanoparticles mixed with NDs [26], can accelerate spin relaxation (Figure 8d). This results in a two-component, nonmonotonic decay of the fluorescence profile (Figure 8e), reflecting spin and charge dynamics, which becomes steeper with increasing concentration of the paramagnetic agent. This technique enabled the quantification of gadoteridol molecules, a commonly used contrast agent for in vivo MR imaging, surrounding each ND, down to a few tens per particle (Figure 8f). This methodology could apply to protocols that consider only spin relaxation [24,25,137,138,139] and thus improve sensitivity. Additionally, biocompatible and functionalized NDs can be internalized in cells [15,16]: the nonmonotonic dependences of fluorescence on laser power, again suggesting coupled spin and charge dynamics, made it possible to study the microenvironment in different cellular compartments [136].

We conclude this section by noting that charged impurities exist in natural or synthetic diamonds as defect centers that originate during the synthesis or the implantation. Some of the common defects include nitrogen composites, boron, single and multiple vacancies, and silicon-vacancy (SiV) centers. Owing to the sensitivity to an electrostatic potential, the NV charge dynamics can be exploited to identify and monitor these defect centers in diamond. For instance, using single-point irradiation and multilaser diamond rastering, the charged states of SiV have been determined [140,141]. Additionally, the diffusion coefficients of electrons and holes in diamond and the trapping constants of substitutional nitrogen, NV centers, and SiV centers have been recently extracted [140,142]. Interestingly, with the same strategy, the existence of an optically and magnetically dark defect (presumably related to hydrogen) was discovered [143].

### 5.2. Applications That Require Stabilization of Charges

Most applications using NV^−^ centers, such as magnetometry and thermometry, rely purely on their spin. Operating with NV^−^ requires optical and microwave radiation pulses to prepare, manipulate, and read the spin states. It is crucial that the NV^−^s charge state is stable, both under such manipulations and in the dark, since charge conversion can deteriorate the signal in two ways: (1) the NV^−^→ NV^0^ conversion reduces the number and the FL intensity from NV^−^ centers with respect to the NV^0^ signal; (2) the NV^0^→ NV^−^ conversion creates a pool of spin-uninitialized NV^−^ centers that reduce the signal-to-noise (SNR) ratio. By way of example, Yuan et al. [38] and Gorrini et al. [35] pointed out a reduction in the ODMR contrast for shallow NV^−^ centers caused by charge instabilities at the surface. Hence, these phenomena affect the sensitivity η to magnetic fields. For a Ramsey scheme and with an NV^−^ ensemble, the DC magnetic sensitivity reads [76]:

(3) $$\eta_{ensemble}^{Ramsey}\approx \frac{\hbar }{\Delta m_{s}g_{e}\mu_{B}}\frac{1}{\sqrt{N\tau }}e^{{\left(t/T_{2}^{*}\right)}^{p}}\sqrt{1+\frac{1}{C^{2}n_{avg}}}\sqrt{\frac{t_{I}+\tau +t_{R}}{\tau }}$$

The first term corresponds to the spin projection noise, *ℏ* is the Planck constant, $\Delta m_{s}$ the difference in spin quantum number between two interference states (such as 0 and −1), $g_{e}$ is the *g* factor, $\mu_{B}$ is the Bohr magneton, and *N* and τ represent the number of NV^−^ centers and the interrogation time, respectively. The second limiting term considers spin dephasing and is characterized by $T_{2}^{*}$. The coefficient *p* depends on the most common type of impurity affecting the dephasing; p=1 for dipolar–dipolar interactions among NV^−^ (high-density limit) and p=3 for dipolar coupling to other spins, such as ^13^C [144]. The third term depends on the readout fidelity, and it is described by the contrast *C* and the average number of photons $n_{avg}$. Finally, the last term considers an overhead time due to the initialization and read-out pulses ($t_{I}$ and $t_{R}$), which extend the duty cycle without improving readout efficiency. Clearly, the contrast, $n_{avg}$, and the dephasing time are affected by the instability of charges and defect states. It has been shown that optimizing the density of NV^−^ centers by synthesis, nitrogen implantation, and laser irradiation greatly improves sensitivity [35,66,145,146].

We emphasize that NV-based magnetometry is a highly flexible technique, adaptable to a large variety of biosystems, both in vitro and in vivo. By way of example, Barry [147] measured the action potential, with single-neuron sensitivity, of the marine fanworm Myxicolainfundibulum and the North Atlantic longfin inshore squid Loligopealeii, while Webb [148] measured the magnetic field in a living mouse muscle optogenetically activated with blue light. Other studies have involved the field produced by magnetotactic bacteria [149], iron biomineralization in chiton teeth [150], and the dynamics of cells containing NDs through translation and rotation tracking of NV centers [151,152]. Similar conclusions hold for other detection sequences used in biosensing, such as continuous or pulsed ODMR for DC fields; Hahn echo or CPMG for AC fields; and even sequences used for thermometry, electrometry, and detection of pressure and strain fields and nuclear spins, as long as sensitivity depends on a spin-based FL contrast.

**Figure 9:**
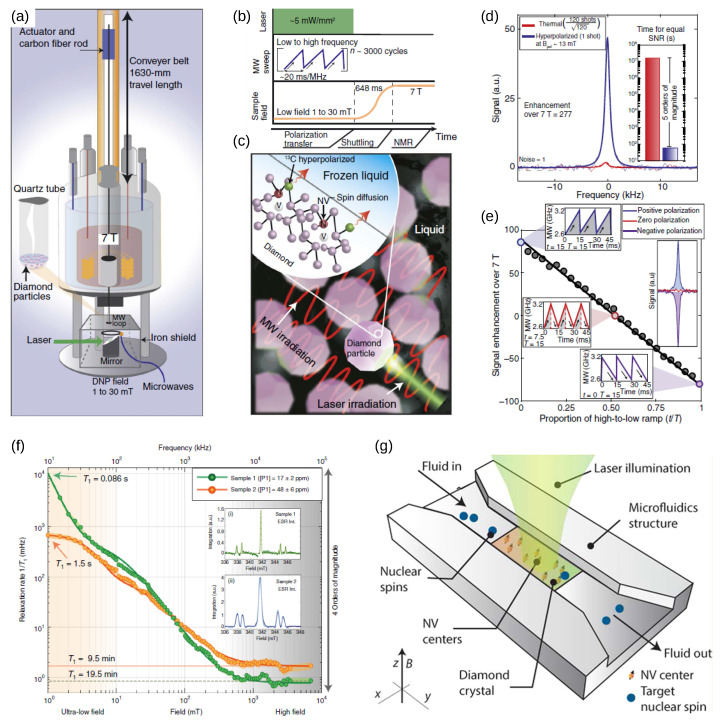
A sketch of a hyperpolarization setup (**a**), comprising a laser, a microwave loop, and a tunable magnetic field for a DNP of ${}^{13}$C atoms in microdiamonds (**c**) and a fiber rod to quickly shuttle the system into the high-field region of an NMR apparatus. The protocol used for hyperpolarization, displayed in (**b**), is robust under random ND orientation and is based on fast, partly nonadiabatic traversals of a pair of Landau–Zener crossings [156]. The arising ${}^{13}$C polarization shows a factor 277 enhancement compared with that of thermal polarization at 7 T (**d**) and can be positive or negative depending on the direction of the microwave sweep (**e**). Readapted from Ajoy et al. [156] with permission. Copyright 2018 Science AAAS. As a first application, the ${}^{13}$C nuclear spin-lattice relaxation time is mapped at various magnetic fields in two diamonds with different concentrations of P${}_{1}$ centers (**f**). The laser-driven hyperpolarization is first transferred to ${}^{13}$C nuclei, which then relax at the desired magnetic field before high-field detection. Reprinted with permission from Ajoy et al. [165]. Copyright 2019 Springer Nature. A proposal for a diamond-based device equipped with a microfluidic structure capable of transfering hyperpolarization to external nuclei of a fluid is illustrated in (**g**). Reprinted with permission from Abrams et al. [160]. Copyright 2014 American Chemical Society.

Building a higher density of NV^−^ compared with NV^0^ is also beneficial for ^13^C nuclear hyperpolarization (Figure 9a–e). Many techniques based on the polarization transfer from NV^−^ centers to ^13^C nuclei in the diamond lattice have been developed in recent years. These include purely optical techniques, such as high-field double NV spin-flip [153], and level anticrossing at ground [154] or excited states [155]. Additionally, microwaves-aided dynamic nuclear polarization (DNP) protocols based on the solid effect [30], nonadiabatic level crossing [156], Hartmann–Hahn [157], and PulsePol [158,159] have been developed. The ultimate goal for all these techniques is to transfer polarization to nuclear spins outside the diamond, e.g., to molecules adsorbed at the surface or in the suspension medium, typically in the form of a fluid or a crystallized solution (Figure 9g) [159,160,161]. Other hyperpolarization techniques have been proposed as a means to dramatically increase the sensitivity of NMR and MRI up to five orders of magnitude, including dynamic nuclear polarization, para-hydrogen-induced polarization, optical pumping, and spin exchange (reviewed in [162]). However, all these methods present limitations that have so far hampered their widespread use in biomedicine, including the need for potentially harmful radicals and complex cryogenic apparatus. Conversely, diamond has an excellent safety and biocompatibility profile, and NV centers embedded in the diamond lattice can be polarized at room temperature, thus providing an ideal route for the hyperpolarization of nuclear spins. However, despite intense investigation, substantial polarization of nuclear spins has been proven difficult to obtain, possibly due to the depletion of NV^−^ centers at the diamond interface in favor of the more stable NV^0^.

Two strategies of spin transfer to nuclear spins outside the diamond have been envisioned: the first is via direct polarization of external ^13^C atoms by shallow NV centers. Needless to say, this strategy requires stable shallow NV^−^s, which in turn demand a recharge of NV^0^ centers and a suppression of shallow and surface acceptor states. Additionally, recharging the NV^0^ has the additional advantage of suppressing their spin-½-induced noise, which could contribute as a relaxation channel for ^13^C nuclear polarization.

The second strategy is via hyperpolarizing the first or second neighbor carbon atoms and then exploiting spin diffusion from the ^13^C spin reservoir through the diamond surface (Figure 9c). The buildup of a bulk polarization is expressed by the equation [161]:

(4) $$\frac{\partial P\left(r,t\right)}{\partial t}=u\left(r\right)\left[1-P\left(r,t\right)\right]-\Gamma_{1,n}P\left(r,t\right)+D_{n}\nabla^{2}P\left(r,t\right)$$

where $P\left(r,t\right)$ is the ^13^C nuclear polarization at distance *r* from the NV center and time *t*, $u\left(r\right)$ is the position-dependent polarization rate, which depends on flip-flop processes (Schwartz 2018), $D_{n}$ is the nuclear spin diffusion coefficient, and the nuclear spin-lattice relaxation rate $\Gamma_{1,n}$ is the inverse of the spin-lattice relaxation time $T_{1,n}$. The polarization propagates through diffusion, extending from the source until it reaches a steady state that is identified by a diffusion length *L*. The reported values of $D_{n}$ range from ≈1 to ≈10 nm^2^/s, depending on the magnetic field, level of ^13^C enrichment and presence of other defects [163,164,165]. Thus, considering a ^13^C relaxation time of 1–10 s (at the common magnetic fields of a few tens of micro Tesla, Figure 9f), the diffusion length can hardly exceed a few nanometers [165]. This figure should be compared with that of the the NV^−^ nearest-neighbor average distance $\langle r\rangle \approx 0.55\;{\left(\rho_{NV}\right)}^{-1/3}$, returning 10 nm of separation for 1 ppm of NV^−^ density $\rho_{NV}$. For this reason, increasing the density of charge-stable negative NVs might prompt spin diffusion and substantially improve the efficiency of the hyperpolarization process.

## 6. Conclusions

In conclusion, we described in detail the mechanisms that affect charge stability and dynamics in shallow ensembles of nitrogen-vacancy centers in diamond. We argue that the charge switching of NVs in the vicinity of the surface has a strong impact on sensing applications, which rely on shallow NVs for the detection of electric and magnetic fields in the microenvironment surrounding the diamond-based material (e.g., nanodiamonds). While charge dynamics in the bulk are well understood, different mechanisms related to surface states and defects can play a role in nanostructured diamond, typically used for sensing applications in biology and medicine. Importantly, we summarized the strategies used to stabilize or manipulate shallow NVs charge states to improve sensitivity and resolution in bioassays. We showed that NV charge switching can be harnessed to improve spatial resolution in fluorescence microscopy, promoting the development of super-resolution microscopy techniques with strongly fluorescent nanodiamonds. Moreover, we discussed the detrimental role that charge instability may play in spin-polarization-transfer techniques, e.g., for the hyperpolarization of nuclear spins adsorbed at the diamond surface. This detailed description of the underlying physical mechanisms and a summary of techniques that can prevent charge conversion in shallow NVs should promote progress in this area, which has attracted considerable interest for its potential application in biomedicine but has proven frustratingly elusive.

## Data Availability

This is a review paper and no original data have been produced and made available.
